# Liposomes targeting the cancer cell-exposed receptor, claudin-4, for pancreatic cancer chemotherapy

**DOI:** 10.1186/s40824-023-00394-7

**Published:** 2023-05-26

**Authors:** Chaeeun Bang, Min Gyu Park, In Kyung Cho, Da-Eun Lee, Gye Lim Kim, Eun Hyang Jang, Man Kyu Shim, Hong Yeol Yoon, Sangmin Lee, Jong-Ho Kim

**Affiliations:** 1 College of Pharmacy and Bionanocomposite Research Center, Kyung Hee University, Seoul, 02447 Republic of Korea; 2 Biomedical Research Institute, Korea Institute of Science and Technology, Seoul, 02797 Republic of Korea; 3 Department of Regulatory Science, Graduated School, Kyung Hee University, Seoul, 02447 Republic of Korea

## Abstract

**Background:**

Claudin-4 (CLDN4), a tight junction protein, is overexpressed in several types of cancer, and is considered a biomarker for cancer-targeted treatment. CLDN4 is not exposed in normal cells, but becomes accessible in cancer cells, in which tight junctions are weakened. Notably, surface-exposed CLDN4 has recently been found to act as a receptor for *Clostridium perfringens* enterotoxin (CPE) and fragment of CPE (CPE17) that binds to the second domain of CLDN4.

**Methods:**

Here, we sought to develop a CPE17-containing liposome that targets pancreatic cancers through binding to exposed CLDN4.

**Results:**

Doxorubicin (Dox)-loaded, CPE17-conjugated liposomes (D@C-LPs) preferentially targeted CLDN4-expressing cell lines, as evidenced by greater uptake and cytotoxicity compared with CLDN4-negative cell lines, whereas uptake and cytotoxicity of Dox-loaded liposomes lacking CPE17 (D@LPs) was similar for both CLDN4-positive and negative cell lines. Notably, D@C-LPs showed greater accumulation in targeted pancreatic tumor tissues compared with normal pancreas tissue; in contrast, Dox-loaded liposomes lacking CPE17 (D@LPs) showed little accumulation in pancreatic tumor tissues. Consistent with this, D@C-LPs showed greater anticancer efficacy compared with other liposome formulations and significantly extended survival.

**Conclusions:**

We expect our findings will aid in the prevention and treatment of pancreatic cancer and provide a framework for identifying cancer-specific strategies that target exposed receptors.

**Graphical Abstract:**

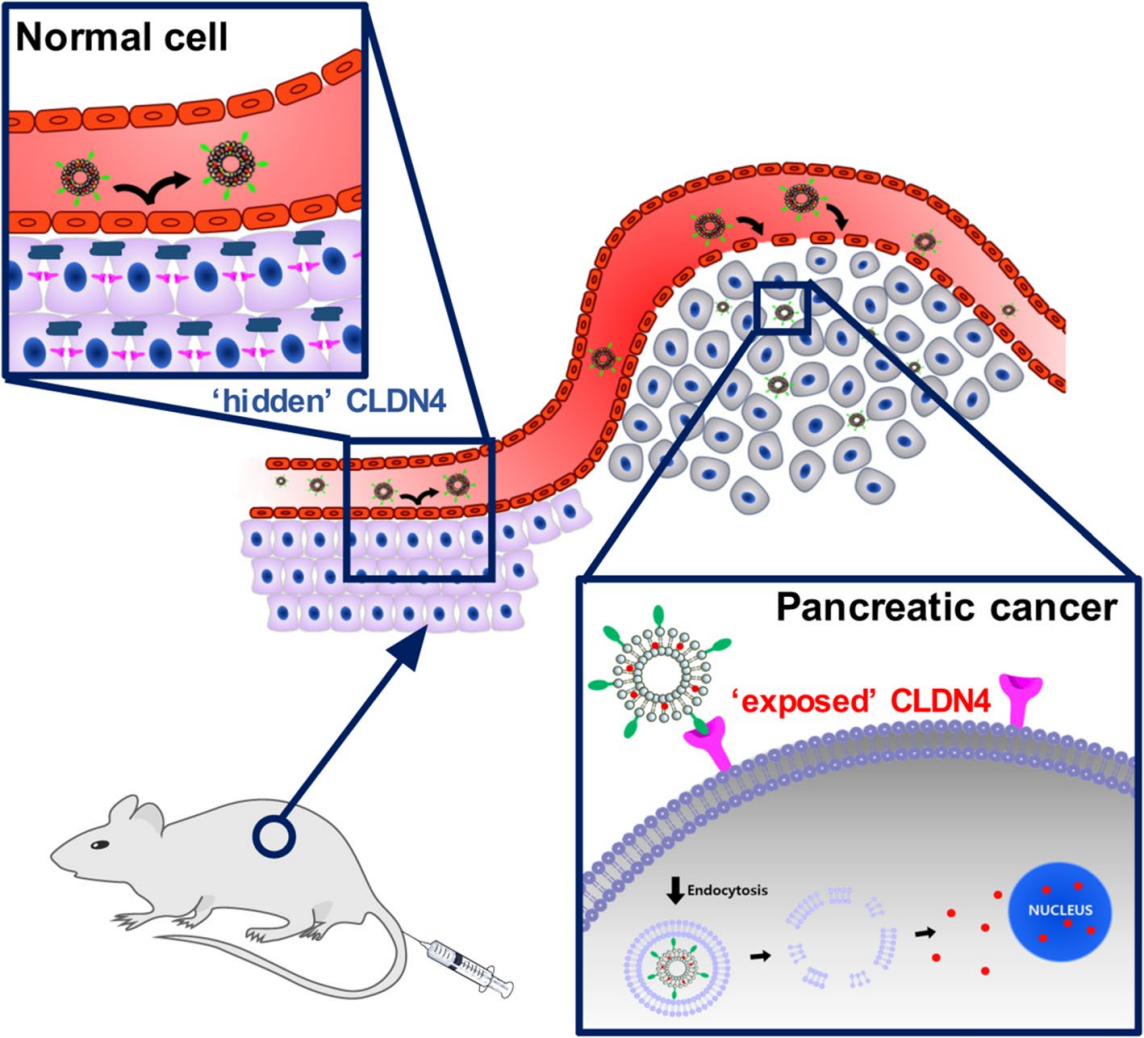

**Supplementary Information:**

The online version contains supplementary material available at 10.1186/s40824-023-00394-7.

## Background

Pancreatic cancer is currently the fourth-leading cause of cancer-associated mortality and is projected to be the second-leading cause of cancer-related death within the next decade [1]. This disease is so aggressive that the tumors of a majority of patients are unresectable at the time of diagnosis. Moreover, more than 80% of patients who undergo surgical resection relapse within 2 years [2]. Despite many recent clinical trials of conventional chemotherapeutic strategies employing single agents or combinations of agents, survival outcomes remain poor.

There has recently been a paradigm shift in the therapy of many cancers from traditional cytotoxic chemotherapies to more targeted therapies; this has led to improved anticancer efficacy and reduced toxic side effects [3, 4]. However, an effective targeted chemotherapy for pancreatic cancer has not yet been developed, highlighting the urgent need for novel therapeutic strategies that reduce the mortality of pancreatic cancer patients. The biggest hurdle in the development of successful targeted chemotherapies based on specific ligand-receptor binding is identification of the appropriate receptor on the surface of tumor cells or tissues [5–7]. A number of such cancer cell-associated receptors, including α_v_β_3_ integrin, epidermal growth factor receptor (EGFR), vascular endothelial growth factor receptor (VEGFR) and folate receptor, have been utilized in ligand-receptor binding systems to improve targeted chemotherapy for pancreatic cancer [8–12]. However, off-target effects reflecting expression of receptors in normal cells still remain a concern for clinical applications [13, 14].

Using a bio-orthogonal click chemistry approach, we previously suggested a strategy for expressing and targeting an artificial azide-group receptor in tumor cells that is normally absent in vivo [15–17]. Here, we suggest another ligand-receptor binding strategy for targeted chemotherapy that utilizes claudin-4 (CLDN4), a receptor that is inaccessible in the normal environment but is exposed in a tumor environment. Members of the CLDN family are important components of tight junctions, which regulate paracellular permeability, cell polarity and barrier function permanence [18, 19], and are closely associated with malignant phenotypes [20, 21]. In normal tissue, CLDNs are hidden since they are located at the most apical component of intercellular cell–cell junctions, which establish cell polarity and regulate cellular diffusion and permeability between apical and basolateral compartments [22]. However, in tumor tissue, CLDNs are exposed because the integrity of tight junctions is lost in tumor cells [23, 24]. This differential CLDN exposure pattern thus offers a promising strategy for specifically targeting receptors in cancer cells [25–27]. Among CLDNs, CLDN4 in particular has been suggested for ligand-receptor binding strategies for improving the efficacy of targeted chemotherapy for pancreatic cancer, as noted above. Interestingly, *Clostridium perfringens* enterotoxin (CPE), a 35 kD polypeptide, is a specific ligand for CLDN4 [28]. In pancreatic cancer studies, a carboxy-terminal fragment of CPE has been widely used for investigating binding of CLDN4 receptors on the surfaces of target cells because full-length CPE has lethal effects [29–32]. However, CPE peptides have not yet been extensively investigated as targeting moieties for nanocarriers.

**Fig. 1:**
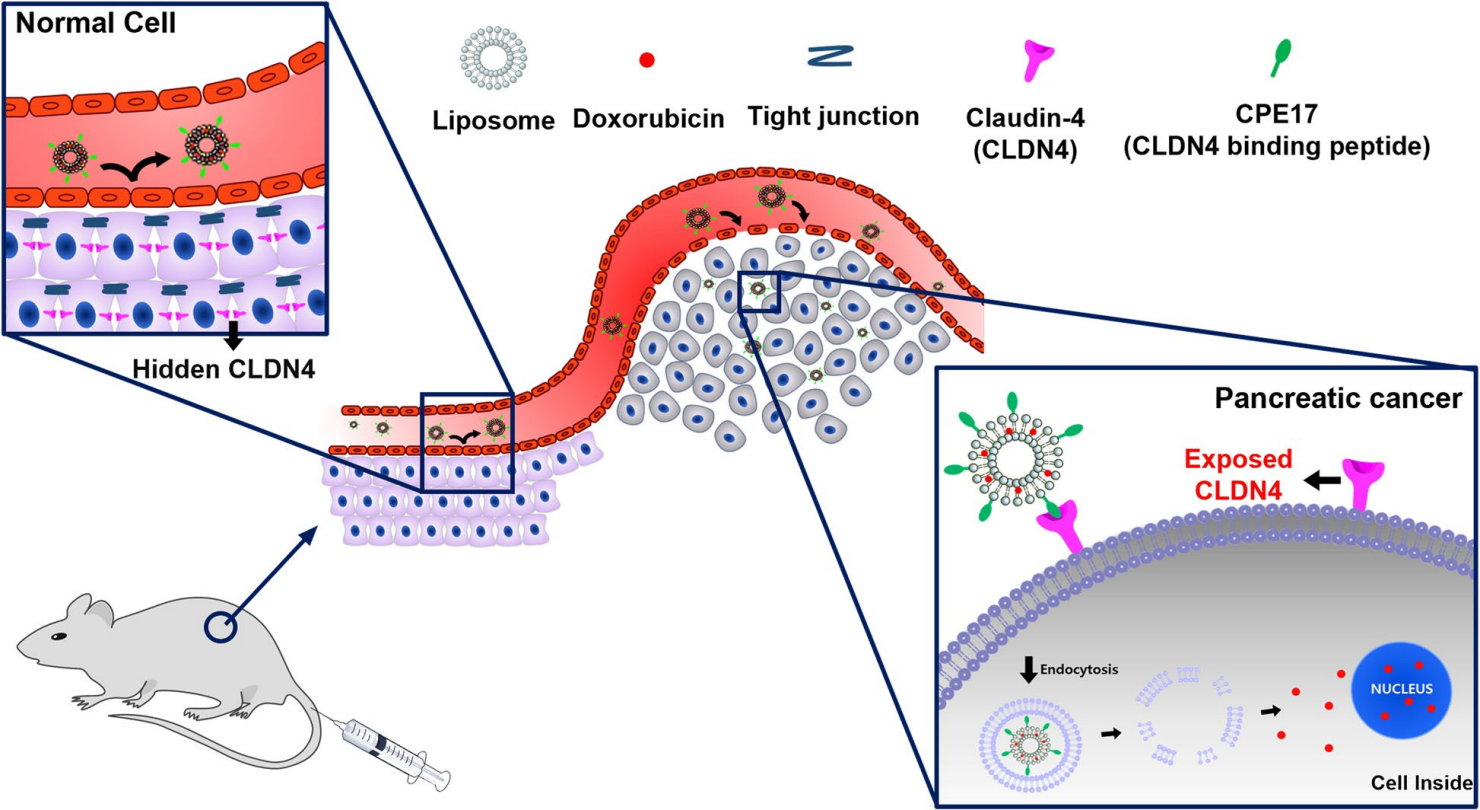
Schematic illustration showing targeting of ‘exposed’ CLDN4 by CPE17-conjugated liposomes for pancreatic cancer chemotherapy. Dox-loaded, CPE17-conjugated liposomes (D@C-LPs) showed extensive accumulation in targeted pancreatic tumor tissues through the EPR effect and targeting of superficially exposed CLDN4, made accessible by disruption of tight junctions. In contrast, accumulation of D@C-LPs in the normal pancreas was significantly reduced because ‘hidden’ CLDN4 in these tissues is sequestered in tight junctions, hindering access to CPE17

Here, we designed a CPE peptide-conjugated, CLDN4-binding liposome for pancreatic cancer-targeted therapy employing a 17-amino acid CPE peptide (CPE17; NSSYSGNYPYSILFQKF). Doxorubicin (Dox)-loaded, CPE17-conjugated liposomes (D@C-LPs) showed extensive accumulation in targeted pancreatic tumor tissues through the enhanced permeability and retention (EPR) effect and targeting of superficially exposed CLDN4 in pancreatic cancer, made accessible by disruption of tight junctions. In contrast, accumulation of D@C-LPs in the normal pancreas was significantly reduced because CLDN4 in these tissues is ‘hidden’ by sequestration in tight junctions, hindering access to CPE17 (Fig. 1). In this study, we demonstrated rationally designed, CLDN4-targeting liposomes that are highly specific to CLDN4 in vitro and in vivo and efficiently deliver Dox by targeting exposed CLDN4 in pancreatic cancer.

## Methods

### Materials

Phospholipids, 1,2-distearoylphosphatidyl-choline (DSPC) and N-hydroxysuccinimide functioned PEG-lipid, with PEG of molar mass 2000 Da covalently attached via a carbamate linkage to 1,2-distearoylphosphatidylethanolamine (DSPE-PEG-NHS), were obtained from Biochempeg Scientific (Watertown, MA, USA). Cholesterol (Chol) and doxorubicin (Dox) were purchased from Sigma-Aldrich (St. Louis, MO, USA). Claudin-4 receptor (CLDN-4R) binding peptide, *Clostridium Perfringens* Enterotoxin (CPE)-17 was synthesized by PEPTRON (Daejeon, Korea). All of the other chemicals and solvents were of analytical grade.

### Preparation and characterization liposomes

To prepare liposome, CPE-17 was conjugated with DSPE-PEG-NHS by simple coupling method, which is called DSPE-PEG-CPE17. Liposomes were prepared by the thin-film hydration method. Briefly, a mixture of DSPE-PEG-CPE17, DSPE-PEG-NHS, Chol, and DSPC (molar ratio 4:1:40:55) was dissolved in chloroform. To prepare Dox loaded liposomes, Dox dissolved with lipid mixture (weight ratio 1:10) in chloroform. Then, the solvent was evaporated using a rotary evaporator N–N series (EYELA, Japan) in a round-bottomed flask for about 40 min and then hydrated in PBS by sonication in a water bath for 10 min to produce a suspension of liposomes. The liposome suspension was extruded 15 times through a pores size of 100 nm polycarbonate membrane (Millipore, USA) with Extruder kit (Avanti Polar Lipids, USA). The size of liposomes, bare liposome (LP), CPE17-conjugated liposome (LP-C), Dox loaded LP (D@LP), and Dox loaded LP-C (D@LP-C) was measured using a DLS (Malvern, UK). The stability of liposomes was measured by observing the size change in serum at 37 °C and in PBS at 4 °C and 25 °C. The release of Dox from Dox loaded liposomes was investigated by dialysis. Briefly, a sample of D@LP and D@LP-C (2 mL, 2 mg/mL) was placed in a dialysis tube (MWCO 1000) and tightly sealed. Then, the dialysis tube was immersed in 20 mL release medium and gently shaken in water bath at 37 °C. Samples were measured at predetermined time intervals from the release medium over a period of 72 h, and these were put back. The concentration Dox was determined by absorption (λ_Ex_ Max 470 nm, λ_Em_ Max 585 nm).

### In vitro targeting ability of liposomes

A549 (human lung cancer, CLDN-4 negative), ASPC-1 (human pancreatic cancer, CLDN-4 positive), and KPC960 (mouse pancreatic cancer, CLDN-4 positive) cells (2 X 10^4^ cells per well) were selected as cell models to confirm the CLDN-4 targeting of Dox loaded liposomes and cultured in DMEM for ASPC-1 and RPMI for A549 and KPC960 medium with 10% fetal bovine serum and 1% streptomycin/penicillin at 37 °C in 5% CO_2_ for 24 h. After a 12 h treatment with Dox loaded liposomes, the cells were rinsed twice with DPBS and fixed for fluorescence microscope (Leica TCS SP8, Leica Microsystems GmbH, Germany) analysis or detached for flow cytometer (Guava easyCyte, Merck Millipore, Germany) analysis.

### Cytotoxicity and in vitro anti-tumor efficacy of liposomes

A549, ASPC-1, and KPC960 cells were seeded at 1 X 10^4^ cells/well in 96-well plate and stabilized for 24 h at 37 °C CO_2_ incubator. After stabilizing, cells were washed with DPBS and cultures for 24 h various concentration of LP, free Dox, D@LP, and D@LP-C. Viability of cells was evaluated using a well-known MTT assay.

### In vivo anti-tumor efficacy of liposomes in allograft flunk model

All experiments with live animals were performed in compliance with the relevant laws and institutional guidelines of Institutional Animal Care and Use Committee (IACUC) in Research Animal Resource Center of Korea Institute of Science and Technology (Approved number: 2017–109). Athymic nude mice (5 weeks old, 20-25 g, male) were purchased from Orient Bio Inc. (Gyeonggi-do, Korea). To prepare KPC960 tumor-bearing mice, allograft flunk model, a suspension of 1 X 10^7^ KPC960 cells in RPMI media (100 µL) was injected into left flanks of mice. For in vivo tumor targeting study, 1 mg/mL Cy5.5-labeled liposomes were administered (200 µL) by intravenous injection when the tumors grew to approximately 100 mm^3^ in volume. After injection, all time point fluorescence in tumor was measured by IVIS Lumina Series III (PerkinElmer, USA). To evaluate the anti-tumor efficacy of D@LP-C, KPC960 tumor-bearing mice were prepared. Mice were divided in four groups: (i) normal saline, (ii) free Dox at 2 mg/kg, (iii) D@LP at equivalent free Dox amount, and (iv) D@LP-C at equivalent free Dox amount (4 mice per group). When tumors reached 100 mm^3^ in volume, each sample was injected once every three days. Tumor size were calculated as *a* X *b*^2^/2, were *a* was the largest and *b* the smallest diameter. After treatment for 15 days, mice were sacrificed to investigate ex vivo analysis. Major organs and the tumors were excised and observed using the IVIS Lumina Series III and fluorescence intensities were calculated using the Living Image software. Excised tissues were fixed 3% paraformaldehyde solution and embedded in paraffin. Embedded tissues were sectionalized for 8 mm using a Cycle Type Paraffin Section Machine (QP3268, Changzhou Haosilin Medical Instrument Co., Ltd., China). After section of tissues, the tumors were treated with TUNEL assay and major organs were treated with H&E assay, which were observed by Leica light microscope.

### Orthotopic implantation of KPC960 cells

The five weeks old male Balb/c nude mice were anesthetized by anesthetic drug (Rompun: Zoletil: saline solution = 1: 4: 45 in 10 mL). A small incision (2 cm) was made in the left abdominal wall. The spleen was then exteriorized along with the underlying pancreas and KPC960 cells (1 X 10^5^ cells per mouse) suspended in media were slowly injected directly into the pancreas. Then, the wound was closed with 6–0 non-absorbable sutures.

### In vivo anti-tumor efficacy of liposomes in orthotopic model

After 7 days of implantation, mice were randomly grouped (*n* = 8): i) normal saline, (ii) free Dox at 2 mg/kg, (iii) D@LP at equivalent free Dox amount, and (iv) D@LP-C at equivalent free Dox amount. Mice were injected drugs intravenously every three days except for weekend. Following the fifth administration (day 24), ex vivo imaging including major organs and tumors was conducted. The weight of the imaged tumor, primary and metastatic tumor located in peritoneal cavity, was measured to make graph (*n* = 3). After fixation and section of tissues, histological assays, colocalization of liposomes with CLDN and TUNEL assay, were performed to confirm the anti-tumor efficacy. In addition, major organs were treated with H&E assay. The colocalization was examined by fluorescence microscope and others were observed by Leica light microscope. Except for the mice that conducted ex vivo, the rest of the mice observed the survival time (*n* = 5).

### Statistical analysis

In this study, the differences between control and experimental groups were analyzed using one-way ANOVA and considered statistically significant marked with asterisk (*p < 0.05, **p < 0.01) in the figures.

## Results

### Preparation and characterization of liposomes

**Fig. 2:**
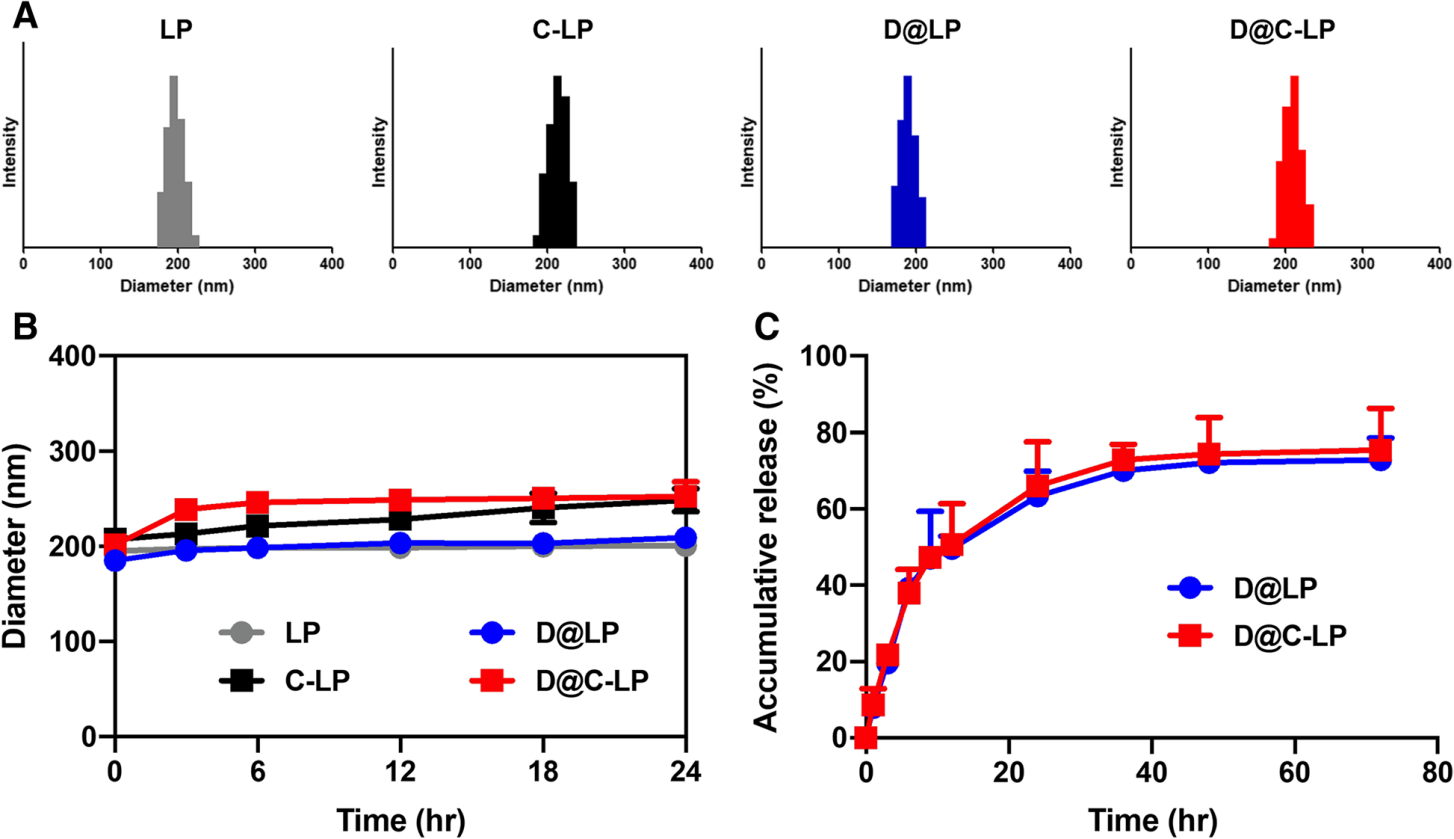
Characterization of liposomes. **A** Size distribution of liposomes. **B** Stability of liposomes, measured as changes in liposome size in serum at 37 °C. **C** Release profile of Dox from D@C-LPs in PBS (pH 7.4) at 37 °C. Error bars represent standard deviations (*n* = 5)

We prepared bare liposomes (LPs), CPE17-conjugated liposome (C-LPs), Dox-loaded LPs (D@LPs) and Dox-loaded C-LPs (D@C-LPs). Liposomes were approximately 200 nm in size and possessed a narrow polydispersity index (PDI) (Table 1, Fig. 2A). They were also well dispersed in saline and showed no change in size after incubating for 1 day at 37 °C in human serum, suggesting their stability under physiological conditions (Fig. 2B). We also investigated liposome stability during storage at room temperature and with refrigeration by examining changes in the size of liposomes incubated in phosphate-buffered saline (PBS) at 4 °C or 25 °C for 1 week. As expected, D@C-LPs were stable, as evidenced by the absence of changes in liposome size and PDI (Figure S1). To assess the potential of our liposomes as drug carriers, we evaluated the release profile of Dox from liposomes at 37 °C in PBS (pH 7.4). Dox showed sustained release from both LP and C-LP preparations (Fig. 2C), a profile typical of drug-loaded liposomes.

**Table 1:** Physicochemical characteristics of the C-SNPs and DOX-C-SNPs

**Material**	**Diameter** **[nm]**	**PDI**^**a**^^**)**^	**Loading efficiency** **[%]**
LP	196.5 ± 5.0	0.097	-
C-LP	215.3 ± 3.2	0.091	-
D@LP	190.1 ± 2.1	0.142	87 ± 3
D@C-LP	209.6 ± 2.3	0.109	88 ± 2

^a^^)^*PDI* polydispersity index

### CLDN4-dependent cytotoxicity and cellular uptake of Dox-loaded liposomes

**Fig. 3:**
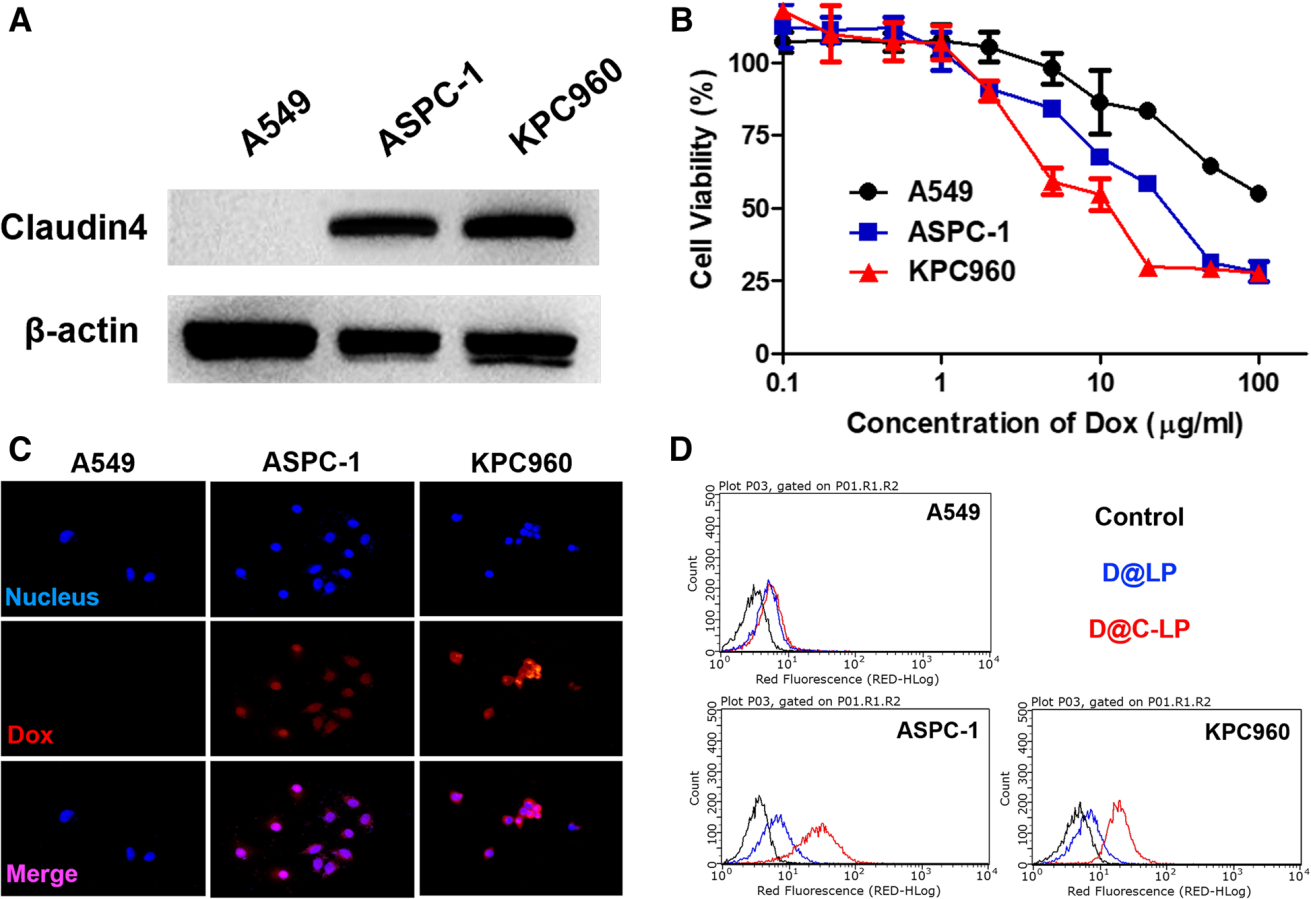
**a** Intracellular drug-release behavior of DOX-C-SNPs, observed by confocal laser-scanning microscopy after a 24 h incubation, compared with that of free DOX. **b** In vitro cytotoxicity of DOX-C-SNPs, DOX-SNPs and free DOX towards KPC960 cells after incubation for 24 h. Data are presented as means ± SDs (*n* = 5). **c** Cellular uptake of DOX-C-SNPs. KPC960 cells were treated with DOX-C-SNPs, with or without CPE pretreatment. After 24 h, the fluorescence of Cy5.5 (red) was measured by confocal laser-scanning microscopy. Scale bar: 50 nm. Nuclei were counterstained with DAPI (blue). Representative flow cytometry data are presented in (**d**)

To investigate targeting of liposomes to cell surface-expressed CLDN4 *in vitro*, we assessed the cytotoxicity of D@C-LPs in three different cancer cell lines that differentially express CLDN4: ASPC-1 and KPC960 cells, which express CLDN4, and A549 cells, which are negative for CLDN4 expression (Fig. 3A). To examine the cytotoxicity of liposomes, we first incubated the three cancer cell lines with free Dox, D@LPs, or D@C-LPs at different Dox concentrations (Figure S2). As expected, the cytotoxicity of D@C-LPs towards cancer cells varied according to the expression of CLDN4, with more severe cytotoxicity observed in CLDN4-positive ASPC-1 and KPC960 cells compared with CLDN4-negative A549 cells (Fig. 3B). Next, we assessed CLDN-dependent cellular uptake of D@C-LPs (5 mg/ml Dox equivalent) against cancer cells by evaluating intracellular Dox fluorescence by fluorescence microscopy after incubating cells with D@C-LPs for 12 h. As shown in Fig. 3C, Dox (red) was localized in the nucleus (blue) of ASPC-1 and KPC960 cells, indicating that D@C-LPs were taken up by CLDN4-positive cancer cells and subsequently released Dox intracellularly. In contrast, no nuclear Dox was detected in CLDN4-negative A549 cells. To more quantitatively assess D@C-LP uptake and Dox release, we performed flow cytometry analyses (Fig. 3D). After incubating cancer cells with D@LPs or D@C-LPs for 4 h, the fluorescence intensity of CLDN4-positive ASPC-1 and KPC960 cells was higher with D@C-LP treatment than with D@LP treatment, whereas the fluorescence intensity of CLDN4-negative A549 cells was similar with D@C-LP and D@-LP treatment. These data indicate that D@C-LPs are highly specific for CLDN4-positive cancer cells in vitro, strongly validating our rationally designed CLDN4-targeting liposomes.

### In vivo therapeutic efficacy of Dox-loaded liposomes in allograft model

**Fig. 4:**
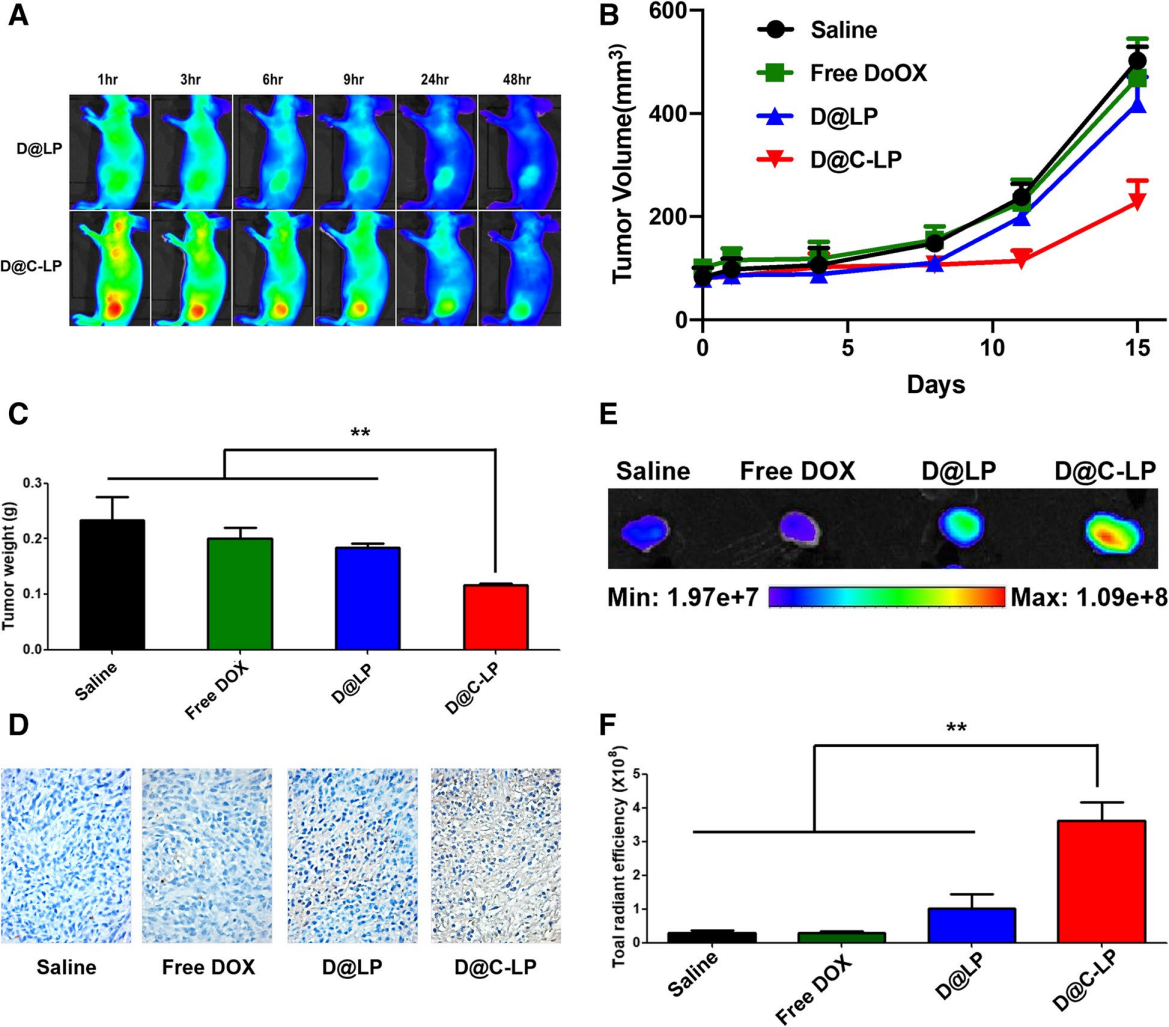
In vivo therapeutic efficacy of Dox-loaded liposomes in a KPC960 xenograft model. **A** In vivo biodistribution of Cy5.5-labeled liposomes. **B** Changes in tumor volume with a 15-day treatment. **C** Average weight of excised tumors after a 15-day treatment. **D** Apoptotic cell death after a 15-day treatment, assessed by TUNEL assay. **E** Ex vivo findings showing fluorescence images of tumors excised from mice treated with Cy5.5-labeled liposomes after a 15-day treatment. **F** Quantification of ex vivo results (*n* = 4). (*P < 0.05, **P < 0.01)

To examine the tumor-targeting ability of C-LPs in vivo, we intravenously injected Cy5.5-labeled D@LPs or D@C-LPs into athymic nude mice bearing CLDN4-positive KPC960 tumors and monitored the in vivo biodistribution of liposomes over time (Fig. 4A). Near-infrared fluorescence (NIRF) microscopy revealed signals throughout the body of mice within 1 h of intravenously injecting either D@LPs or D@C-LPs, indicating that both liposomes circulated rapidly in the blood. D@C-LP NIRF signals at the tumor site were stronger than those of D@LPs, indicating much greater accumulation of D@C-LPs in tumors compared with D@LPs and supporting strongly enhanced targeting ability of C-LPs compared with LPs in this CLDN4-positive allograft model. To further demonstrate the selectivity of D@C-LP, pre-treatment of CLDN4 antibody performed for in vivo biodistribution in CLDN4-positive xenograft model. As we expected, pre-treatment of antibody inhibited the accumulation of liposomes in tumor (Figure S3). Again using the KPC960 tumor-bearing allograft model, we next evaluated the antitumor efficacy of D@C-LPs. After tumors reached a volume of ~ 200 mm^3^, mice were injected with one of the following four formulations every 3 days: (i) normal saline, (ii) free Dox at 2 mg/kg, (iii) D@LPs at 20 mg/kg, or (iv) D@C-LPs at 20 mg/kg. The therapeutic efficacy of liposomes was assessed by measuring tumor volumes over the course of 15 days (Fig. 4B). Fifteen days after injection of liposome formulations, the average volume of tumors in mice treated with D@C-LPs reached just 220 mm^3^, whereas the volume of tumors in other groups was greater than 420 mm^3^. As shown in Fig. 4C, weights of tumors excised from D@C-LP–treated mice were less than those from any other group. These findings are consistent with tumor volume results and are indicative of treatment-dependent antitumor efficacy (Figure S4A). A histological analysis of tumor tissue by TUNEL (terminal deoxynucleotidyl transferase dUTP nick-end labeling) staining revealed severe cell death in the D@C-LP treatment group compared with other groups (Fig. 4D). These results imply much greater relative accumulation of D@C-LPs in tumors. This conclusion is supported by quantitative analyses of fluorescent images of excised tumors (Figure S4B), which revealed more than threefold greater accumulation of D@C-LPs (Fig. 4E and F). Mouse weights were not significantly different among groups throughout the duration of the experiment (Figure S4C), suggesting limited off-target effects of D@C-LPs in normal tissues. To further assess the toxicity of D@C-LPs towards non-target organs, we performed hematoxylin and eosin (H&E) staining of liver, lung, spleen, kidney, and heart tissues after treatment. As shown in Figure S4D, there were no histological abnormalities in any of these major organs. Notably, despite the excellent therapeutic efficacy of D@C-LPs, mice treated with this formulation exhibited a histomorphology similar to that of mice treated with normal saline. Collectively, these results demonstrate that a greater amount of D@C-LPs is delivered to pancreatic tumors in a xenograft model after intravenous injection, resulting in strong chemotherapeutic efficacy.

### In vivo therapeutic efficacy of Dox-loaded liposomes in orthotropic model

**Fig. 5:**
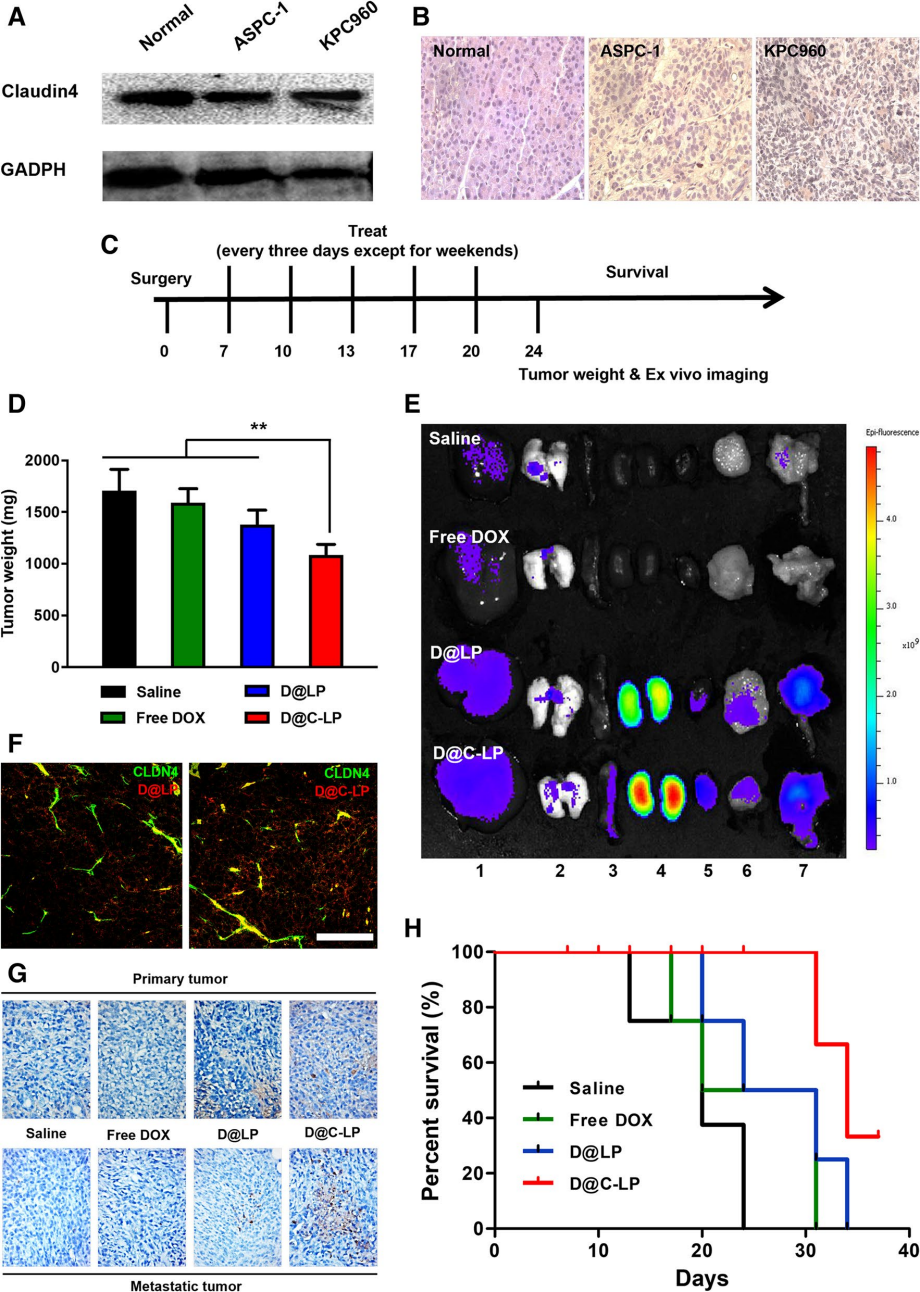
In vivo therapeutic efficacy of Dox-loaded liposomes in a KPC960 orthotopic model. **A** Quantification of CLDN4 expression in normal pancreas and pancreatic cancer tissues by Western blot analysis. **B** Immunohistochemical analysis of normal pancreas and pancreatic cancer tissue sections stained with an anti-CLDN4 antibody. **C** Treatment schedule. **D** Average weight of excised tumor tissues, including primary tumor and metastatic tumor, at 24 days. **E** Fluorescence images of major organs (liver(1), lung(2), spleen(3), kidney(4), and heart(5)) and tumor tissues (primary(6) and metastatic(7) tumors) from the orthotopic model at 24 days. **F** Colocalization of liposomes with DLCN4 in excised metastatic tumor tissue. **G** Apoptotic cell death at 15 days, assessed by TUNEL assay of tumor tissues, including primary tumor and metastatic tumor. **H** Mouse survival over the course of treatment (*n* = 8). (*P < 0.05, **P < 0.01)

Before accepting the apparent efficacy of D@C-LP chemotherapy, it is necessary to confirm the rationale of exposed CLDN4 as a therapeutic target in an orthotopic pancreatic tumor model. To prepare this tumor model, we first anesthetized mice by intraperitoneal (i.p.) injection of ketamine and xylazine, and then performed a laparotomy and directly injected 1 × 10^5^ ASPC-1 or KPC960 tumor cells (in 10 µl of saline) into the pancreas. Ten days after orthotopic implantation of tumor cells in the pancreas, CLDN4 expression in pancreatic tumor tissues was evaluated by Western blot analysis and immunohistochemical staining. Western blot analyses revealed no differences in CLDN4 expression levels between the normal pancreas and pancreatic tumor tissues (Fig. 5A). In contrast, immunohistochemical analyses showed significantly elevated CLDN4 exposure on the surface of cells in pancreatic cancer tissues compared with normal pancreatic tissues (Fig. 5B). These results indicate that, although the overall amount of CLDN4 in pancreatic cancer and normal pancreatic tissue is nearly the same, CLDN4 is superficially exposed in pancreatic cancer compared with the normal pancreas owing to disruption of tight junctions.

Finally, we evaluated the clinical efficacy of D@C-LPs compared with that of saline, free DOX and D@LP by testing these preparations in the KPC960 orthotopic pancreatic tumor model; the DOX concentration in each preparation was 2 mg/kg, and each preparation was intravenously injected once every 3 days from 7 to 20 days after surgery (Fig. 5C). At the end of this period, the average pancreatic primary tumor weight was largest in the normal saline group (799.6 mg), followed by the D@LP group, with an average tumor weight of 689.2 mg. In contrast, treatment with D@C-LPs reduced the average tumor weight on day 20 to 562.0 mg, a substantial decrease in mean tumor weight. Notably, this decrease was significantly greater than that in the free DOX group, where average tumor weight was 685.3 mg (Figure S5A). Interestingly, implanted KPC960 cells gave rise to metastatic tumors in the abdominal cavity in addition to the primary tumor located in pancreas. In investigation of metastatic tumor weight, D@C-LPs showed the best therapeutic efficacy among the groups, where average tumor weigh was 503.6 mg (Figure S5B), which is consistent with primary tumor weight. As shown in Fig. 5D, total tumor weight, including that of the primary tumor and metastatic tumor, was lowest in the D@C-LP–treated group compared with other groups, indicating that D@C-LPs inhibit metastatic tumors as well as primary tumors in this KPC960 orthotopic pancreatic tumor model. Twenty-four days after surgery, major organs were excised and ex vivo fluorescence intensity was measured using an IVIS Lumina system. Ex vivo images of tumor tissues revealed strong fluorescence intensity in tumors—both primary and metastatic—from D@LP– and D@C-LP–treated mice (Fig. 5E). Strong signals were observed in the kindeys, which reflects not the accumulation but renal clearance of liposomes. To support therapeutic efficacy, we observed the colocalization of both liposomes, D@LP and D@C-LP. As we expected, D@C-LP showed more colocalization with CLDN4 compared to D@LP (Fig. 5F). A further histological analysis of tumor tissues by TUNEL staining showed that the positive signal corresponding to apoptotic cells was much stronger in pancreatic tumor tissues from mice treated with D@C-LPs compared with that in tumors (especially metastatic tumors) from mice in all other treatment groups, indicating that D@C-LP treatment effectively induces apoptosis in pancreatic tumor tissues (Fig. 5G). Survival times following treatment with saline, free DOX, and D@LPs were 24, 30 and 33 days, respectively, versus 42 days in the D@C-LP–treated group (Fig. 5H), indicating a substantial survival benefit of D@C-LP treatment.

Before nanoparticles can be applied clinically as anticancer nanomedicines, they must satisfy *in vivo* toxicity requirements. To this end, we measured changes in body weight throughout the survival experiment and performed H&E staining of tissue sections from the major organs, obtained 24 days after surgery. Mouse weights were not significantly different among groups during the experiment (Figure S5C), suggesting that D@C-LPs effectively release the drug in tumors without affecting other normal tissues. Images of H&E-stained tissues showed no significant structural abnormalities or damage in any of the major organs (Figure S5D). This negligible toxicity may be attributable to the low non-specific uptake in normal tissues and high stability of D@C-LPs, demonstrating the potential suitability of D@C-LPs as an anticancer nanomedicine in clinical applications.

## Discussion

As many researchers recognize, effective targeted chemotherapy for pancreatic cancer has not yet been developed and a new therapeutic strategy is urgently needed to reduce the mortality rate of pancreatic cancer. A few nanoparticles containing biological targeting moieties have been recently attempted targeted therapeutic strategies and have obtained significant results. We previously reported that CLDN4-targeting polysialic acid-based nanoparticles could be promising agents for effective pancreatic cancer therapy [33]. CLDN4s show not only upregulation in pancratic tumor tissues but also differential expression patterns between pancreatic cancers and other normal tissues [34, 35]. CPE peptide, CPE17, can bind to exposed CLDN4 on tumor cells while it is difficult to bind hidden CLDN4 on normal cells. Therefore, CLDN4-CPE17 biding is widely expected receptor-ligand system as promising candidate to tumor targeting starategy. Here, a CPE17-conjugated liposome (C-LP) that enables extensive accumulation in targeted pancreatic tumor tissues through the enhanced permeability and retention (EPR) effect and targeting of not hidden but exposed CLDN4 in pancreatic cancer was proposed. From appropriate investigation in vitro and in vivo, doxorubicin loaded CPE17-conjugated liposomes (D@C-LP) shows improved anticancer efficacy and significantly extended survival, which provides a paradime for cancer treatment stargeties targeting cancer cell-specific exposed receptors.

## Conclusions

In this study, we developed CLDN4-targeting *C. perfringens* enterotoxin (CPE)-conjugated liposomes (C-LPs) for pancreatic cancer-targeted therapy. Dox-loaded C-LPs (D@C-LPs) specifically delivered Dox to CLDN4-expressing cancer cells, effectively inducing cytotoxicity. Interestingly, D@C-LPs successfully bound to CLDN4 proteins that had become superficially exposed in pancreatic cancer owing to disruption of tight junctions, but showed reduced accumulation in the normal pancreas because access to CLDN4 in these tissues is hindered by intact tight junctions. By targeting exposed CLDN4, and thus strongly accumulating in pancreatic cancer tissue, D@C-LPs suppressed orthotopic pancreatic cancer growth as well as xenograft pancreatic cancer while causing minimal toxicity to off-target normal tissues. Collectively, our findings suggest the possibility that our CLDN4-targeting D@C-LPs could be promising agents for effective pancreatic cancer therapy.

## Supplementary Information

**Additional file 1: Figure S1. **Storage stability,measured as changes in the size and PDI of D@C-LPs at 4°C and 25°C. **Figure S2.** Cytotoxicity of Dox-loaded liposomes against various cancer cell lines. **Figure S3.** In vivo biodistribution of D@C-LP with pre-treatment of CLDN4 antibody. **Figure S4.** In vivo therapeutic efficacy of Dox-loaded liposomes in a KPC960 xenograft model. **A** Photographs of excised tumors after treatment. **B** *Ex vivo* fluorescence images. **C** Changes in body weight in treatment groups over the course of 15 days. **D** H&E staining of major organsafter 15 days. **Figure S5.** In vivo therapeutic efficacy of Dox-loaded liposomes in a KPC960 orthotopic model. **A** Average tumor weights of excised primary tumors. **B** Average tumor weights of excised metastatic tumors. **C** Changes in body weight in treatment groups over the course of 24 days. **D** H&E staining of major organsafter 24 days.

## Data Availability

All relevant data are available within the article and its supplementary information files, or available from the corresponding authors upon reasonable request.
