# Supplementary material for: Liposomes targeting the cancer cell-exposed receptor, claudin-4, for pancreatic cancer chemotherapy
Source: Biomater Res. 2023 May 26;27:53. doi: 10.1186/s40824-023-00394-7 (PMC10214683; doi:10.1186/s40824-023-00394-7)
Supplement: Supplementary file 1 — Additional file 1: Figure S1. Storage stability,measured as changes in the size and PDI of D@C-LPs at 4°C and 25°C. Figure S2. Cytotoxicity of Dox-loaded liposomes against various cancer cell lines. Figure S3. In vivo biodistribution of D@C-LP with pre-treatment of CLDN4 antibody. Figure S4. In vivo therapeutic efficacy of Dox-loaded liposomes in a KPC960 xenograft model. A Photographs of excised tumors after treatment. B Ex vivo fluorescence images. C Changes in body weight in treatment groups over the course of 15 days. D H&E staining of major organsafter 15 days. Figure S5. In vivo therapeutic efficacy of Dox-loaded liposomes in a KPC960 orthotopic model. A Average tumor weights of excised primary tumors. B Average tumor weights of excised metastatic tumors. C Changes in body weight in treatment groups over the course of 24 days. D H&E staining of major organsafter 24 days. [file 40824_2023_394_MOESM1_ESM.docx]

***Supporting information***

**Liposomes targeting the cancer cell-exposed receptor, claudin-4, for pancreatic cancer chemotherapy**

Chaeeun Bang, Min Gyu Park, In Kyung Cho, Da-Eun Lee, Gye Lim Kim, Eun Hyang Jang, Man Kyu Shim, Hong Yeol Yoon, Sangmin Lee^*^, Jong-Ho Kim^*^


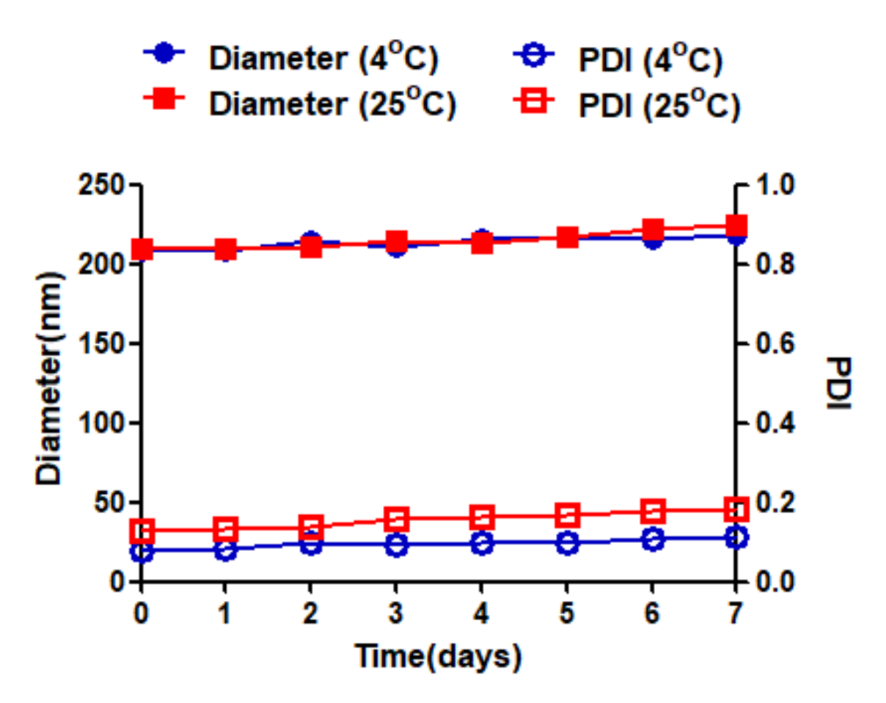


**Figure S1.** Storage stability, measured as changes in the size and PDI of D@C-LPs at 4°C and 25°C.


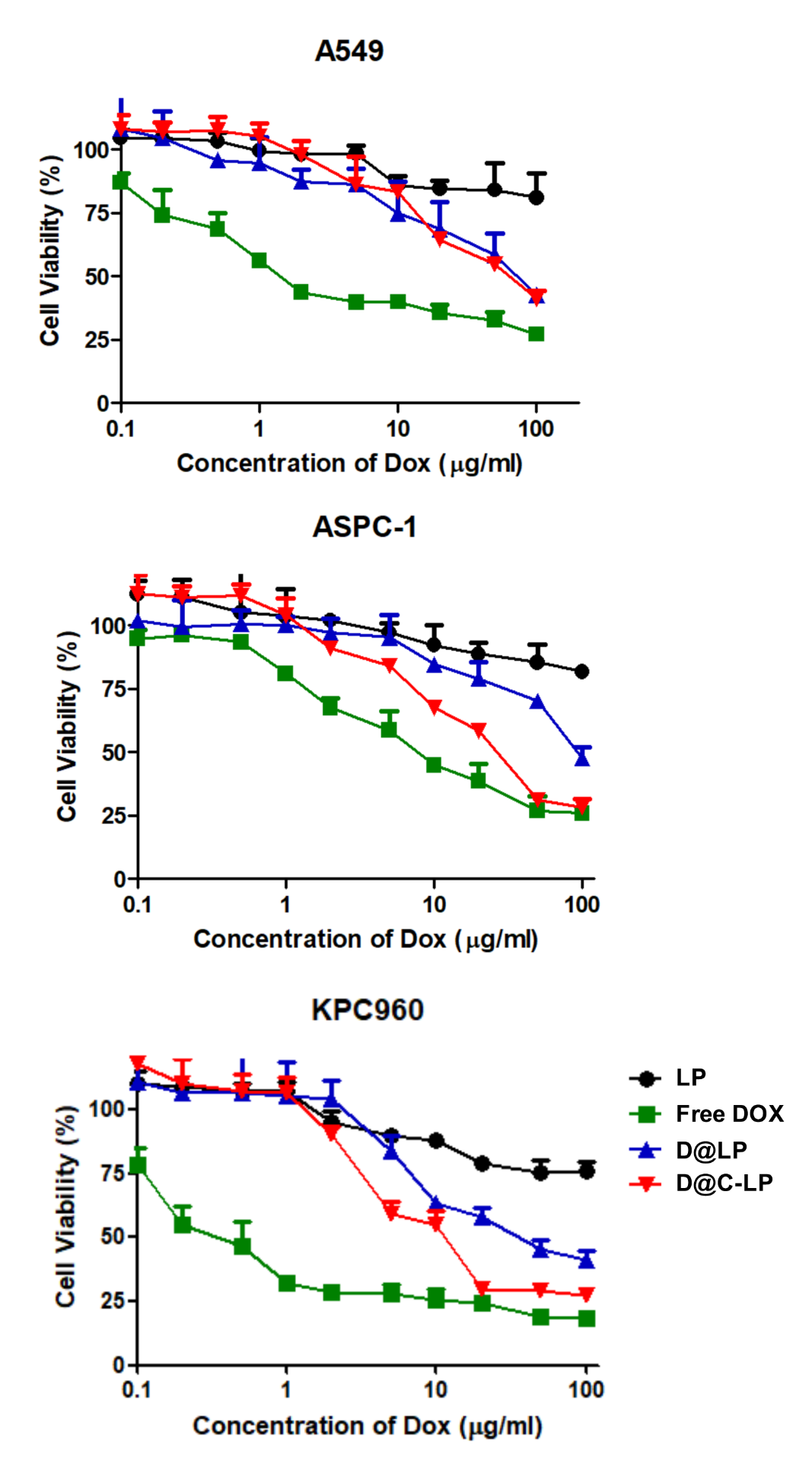


**Figure S2.** Cytotoxicity of Dox-loaded liposomes against various cancer cell lines.


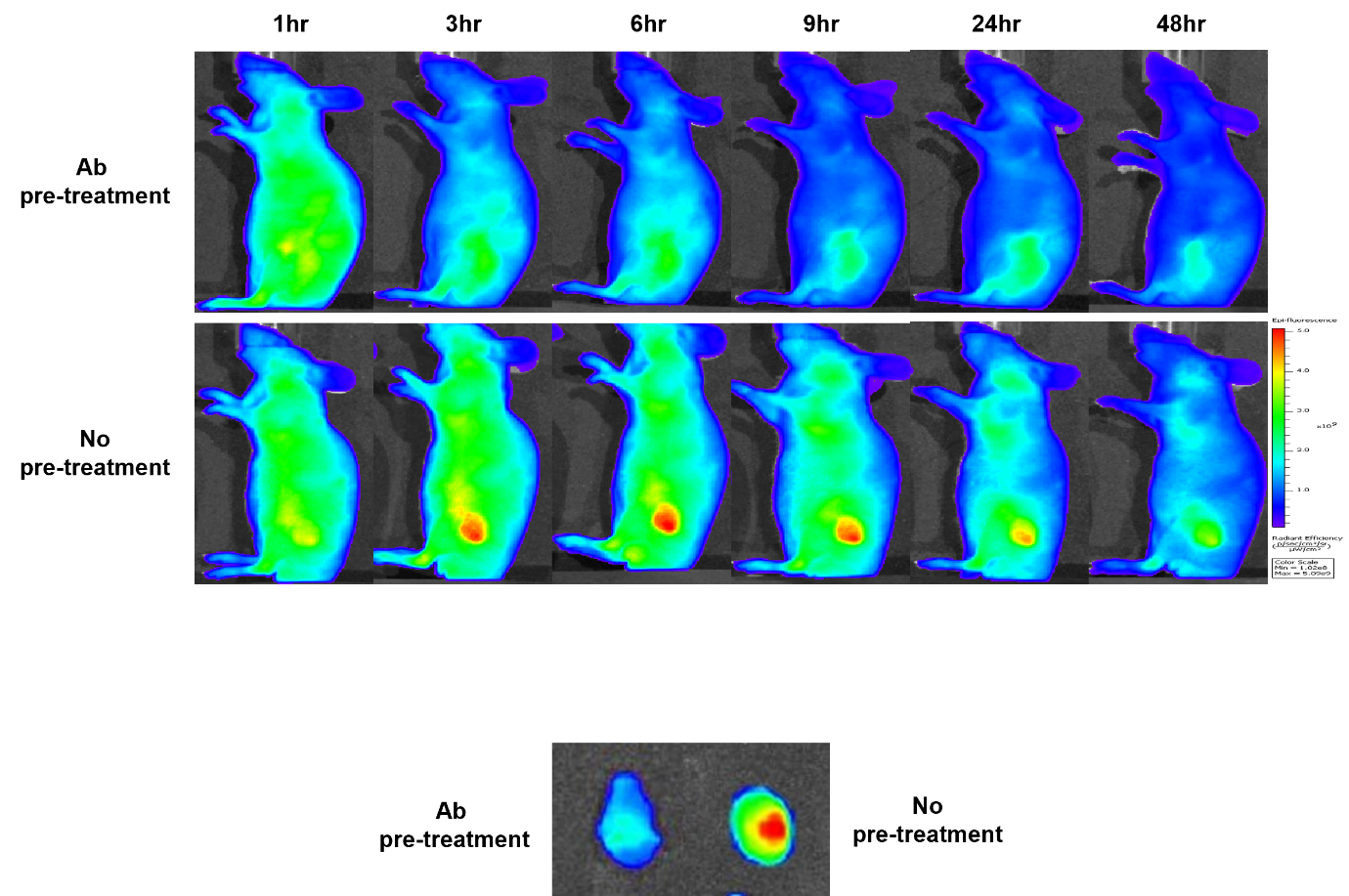


**Figure S3.** *In vivo* biodistribution of D@C-LP with pre-treatment of CLDN4 antibody.


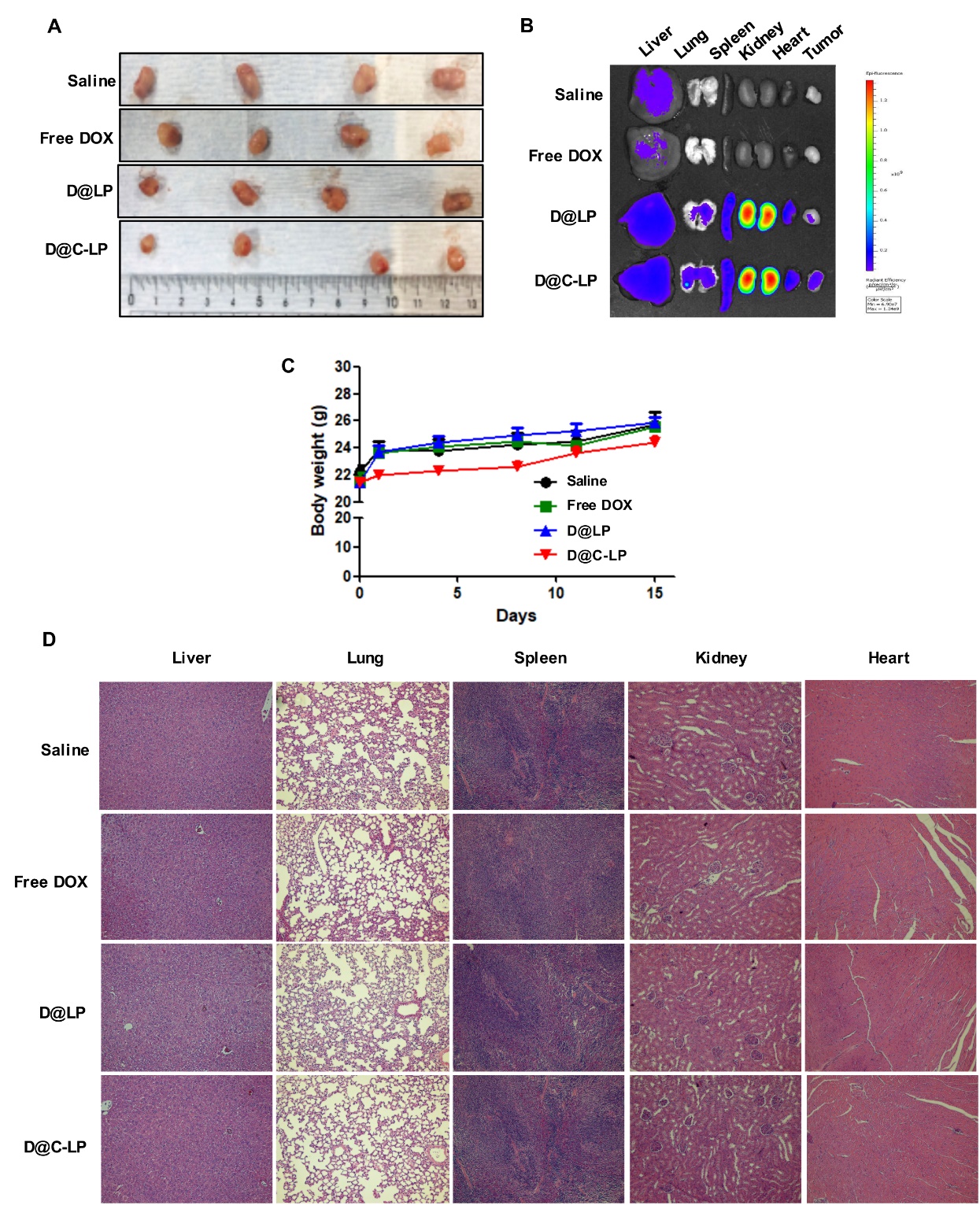


**Figure S4.** *In vivo* therapeutic efficacy of Dox-loaded liposomes in a KPC960 xenograft model. (A) Photographs of excised tumors after treatment. (B) *Ex vivo* fluorescence images. (C) Changes in body weight in treatment groups over the course of 15 days (n = 4). (D) H&E staining of major organs (liver, lung, spleen, kidney, and heart) after 15 days.


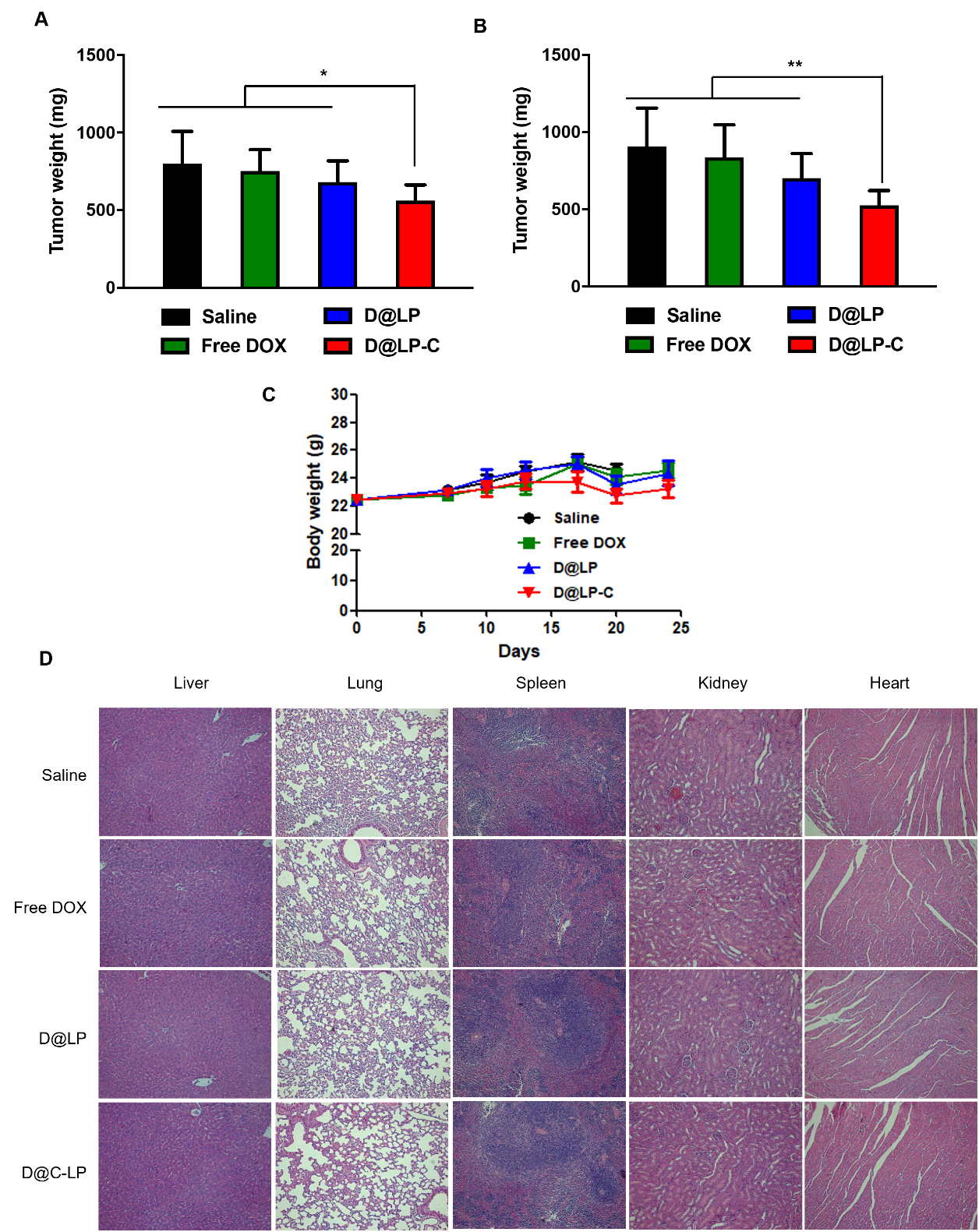


**Figure S5.** *In vivo* therapeutic efficacy of Dox-loaded liposomes in a KPC960 orthotopic model. (A) Average tumor weights of excised primary tumors (n = 8). (B) Average tumor weights of excised metastatic tumors (n = 8). (C) Changes in body weight in treatment groups over the course of 24 days (n = 8). (D) H&E staining of major organs (liver, lung, spleen, kidney, and heart) after 24 days.
